# Hospital and Institutional News

**Published:** 1916-10-28

**Authors:** 


### HOSPITAL AND INSTITUTIONAL NEWS.

### OUR SPECIAL NUMBER.

Next week we shall issue a special number of The Hospital, dealing mainly with hospital architecture and construction. Besides many plans of recent hospital buildings and of additions to existing hospitals, there will appear an exhaustive article on "The Philosophy of Hospital Architecture," by the well-known hospital architect, Mr. William A. Pite, F.R.I.B.A.; and an article on "Military Hospital Construction" by Mr. S. P. Pick, F.R.I.B.A. A problem which is always confronting hospital administrators—namely, the selection of the resident and visiting staffs—will be dealt with in a most instructive paper by Dr. S. S. Goldwater, Superintendent of the Mount Sinai Hospital, New York.

### THE GOVERNMENT AND THE WELSH MEDICAL SCHOOL.

The obstacles which have been placed in the way of the completion of the Welsh Medical School buildings at Cardiff by the Ministry of Munitions were the subject of an emphatic resolution of protest at the last meeting of the Court of Governors of the University College of South Wales. Colonel Bruce Vaughan, that doughty champion of the new Medical School, said at first they experienced great difficulty in convincing the Government that Wales was determined to have a great school, but he was pleased they had at last secured sanction to a scheme which would accommodate at least 250 students. Mr. McKenna offered no objection to the buildings being proceeded with provided the Ministry of Munitions agreed, but, though a deputation went to the Ministry in August and an assurance had been given that no men of military age would be engaged on the buildings, nothing had yet been heard from the Department. Already the delay, according to Colonel Bruce Vaughan, has meant a loss of £15,000 to £20,000 owing to the enhanced price of the materials required. It has been pointed out that if this is to be the treatment meted out, generous citizens like Sir William James Thomas, whose great gift made the Medical School possible, will not feel encouraged in their munificence.

### GARDELEGEN HORRORS.

Of the recently issued official report of the horrors of the typhus epidemic in 1915 at the camp for prisoners of war at Gardelegen, in Germany, it is enough to say that it is a counterpart of that concerning the horrors of Wittenberg. The revolting details—some of them even so horrible that the Government feels unable to print them—of this fresh German outrage on humanity are of the type already familiar. There is no need to repeat them here, but they stand on record for all time as fresh instances of the repulsive barbarity of the modern Germans and of their unfitness to be treated any longer as a civilised nation. How the neutral nations, confronted with these barbarities and outrages, can maintain any semblance of cordial relations with the Huns is a problem which surpasses our comprehension altogether. We cannot imagine anything more calculated to bring the blush of shame to the cheeks of an American or a Scandinavian or a Dutchman than the opportunism of their Governments towards a nation which of set purpose treats its prisoners of war in the way that the unfortunates of Wittenberg and Gardelegen have been treated.

### THE SUCCESS OF A WAR-TIME APPEAL.

The committee of management of King's College Hospital have issued an appeal for £20,000 which, they state, must be raised before the end of the year. Good progress is being made, and at the time of writing some two-thirds of this sum appears to have been promised. The hospital has had an uphill fight financially since its great move to Denmark Hill was undertaken, and it is perhaps doubtful how long such a sum will maintain its income and expenditure in satisfactory proportions. It has been suggested in the newspapers that a wing would have to be closed if the money now asked for were not forthcoming. We hope the fallacious reasoning, so well understood by hospital men, that lies at the back of this worn-out threat, did not emanate from the appeal committee. It is so obvious that at a great institution like King's administration expenses are practically constant whether one part of the hospital be closed or not.

### THE REWARD OF ADMINISTRATION IN LIVERPOOL.

Public Health statistics, if properly compiled, should be the barometer of public health, and it is gratifying to learn that the child welfare work in Liverpool has been accompanied by a pronounced fall in the rate of infantile mortality. It is noteworthy, too, that though the hospital accommodation of Liverpool has been largely drawn upon for military purposes, the demands of civilians upon it appear also to have been much reduced. Whether this state of affairs will be maintained is, of course, doubtful, particularly as the diversion of shipping to Liverpool from other closed ports carries with it an added risk which requires all the care of the Public Health Department to control. Notwithstanding this, the Port Sanitary and Hospitals Committee has been spared such diseases as cholera, plague, and smallpox, and to keep a growing port so free from disease is a tribute to the efficiency of the inspections carried out.

### WAR HOSPITAL MUDDLE AT ASHFORD.

The town of Ashford, in Kent, is much agitated over the question of the conversion of the Technical Institute to the purposes of a war hospital. It seems that the premises hitherto used as a V.A.D. hospital have been condemned as insanitary, and have been ordered to be closed down on December 1. In June last the County Director of the Kent V.A.D. (Lord Chilston) asked for the use of the

Ashford Technical Institute for conversion into a war hospital; and this request was backed up by the A.D.M.S. of the district. The Ashford Higher Education Committee assented in principle, but referred the decision to the County Education Committee. Here the first hitch occurred: the chairman of the higher department of the Kent Education Committee is stated to have hung the scheme up on his own personal initiative. However, in due time this obstacle was surmounted; and the inhabitants of Ashford looked forward to the presence of a fine military hospital in their midst. Long delays followed, during which the V.A.D. authorities had ambitious plans drawn up. Finally, the Board of Education seems to have fixed up a deal with the War Office, behind the backs of the local authorities, and the whole scheme is squashed. Two members of the Ashford Education Committee have resigned as a protest against the action of the Board of Education, and disappointment in Ashford is said to be acute.

### RAWTENSTALL MILITARY HOSPITAL

An addition to the Newhallhey Auxiliary Military Hospital at Rawstenstall has been opened by Colonel W. Coates, of Manchester, with a silver key given by Mr. H. H. Whitehead, J.P. It was announced that the whole of the cost has been defrayed by Lieut.-Colonel J. Craven-Hoyle, of Leabank Hall, Cloughfold; the same donor has also erected a lounge for the men at the hospital, and has promised £50 towards the equipment of the extension. Colonel Coates said that the War Office is ready to accept every hospital offered, and that he is now arranging for an additional five thousand beds. In East Lancashire there are now between four and five thousand beds provided entirely by voluntary effort, apart altogether from those run by the Royal Army Medical Corps. The new extension at Rawtenstall has been named after Lord Kitchener.

### A REMARKABLE VILLAGE COLLECTION.

In the village of Burwell for some years there has been organised a Friendly Societies' Hospital Parade on Feast Sunday, when collections have been made on behalf of Addenbrooke's Hospital, Cambridge. The collection in 1915 exceeded that of previous years, and even that record has been beaten this year. Not being content with this, a collection of gifts in kind was organised in such a systematic manner, thanks largely to the ability of Police-Constable Rowlinson, that about four tons' weight of fruit, vegetables, poultry, eggs, groceries, and so forth were handed to the hospital, and a further consignment of goods to the value of £20 was also forwarded. The success and example of the Burwell Hospital Parade Committee it is hoped will be an incentive for other villages to organise another collection at this season.

### THE CHADWICK TRUST PUBLIC LECTURES.

The programme for the current session of the Chadwick Trust Lectures, which are open free to all, is more definitely concerned with the prevention of disease than usual, and is very comprehensive. Dr. Charles Porter, Medical Officer of Health for Marylebone, is giving three lectures at Norwich on baby-saving, under the title of "The Health of the Future Citizen," on Thursdays at 3 p.m., beginning on November 2. On November 20 Dr. J. T. C. Nash, Medical Officer of Health for Norfolk, tackles much the same subject in a lecture on "Baby-Saving for the Nation." On Fridays at 5.15 p.m., starting on October 27, Dr. William Stirling delivers three lectures on "Fatigue and its Effects on Industry and Efficiency." Lastly, Mr. Paul Waterhouse lectures on November 30 and two subsequent Thursdays on "Architecture in Relation to Health and Welfare." With the exception of Dr. Porter's lectures, these Chadwick Trust Lectures are to be delivered in London at various addresses. Further particulars may be obtained from the Secretary at the offices of the Trust, 40 Queen Anne Chambers, Westminster.

### NAVAL PHILANTHROPY.

The annual meeting of the Royal Naval and Marine Orphan Home, Portsmouth, was held at the Home on Trafalgar Day, October 21, when Admiral the Hon. Sir Stanley C. J. Colville, G.C.V.O., K.C.B., Commander-in-Chief, Portsmouth, presided. The report stated that in a time of great national strain and stress there had been no slackening of interest or sympathy, and amid the many pressing calls of the times, that of the Home had been fully recognised. The satisfactory financial position was due largely to the Trafalgar Fund, which amounted to £4,020 5s. 6d., a considerable increase over any previous year. The sum of £500 had been received from the Sergeants' Mess, Royal Marine Artillery, the result of a camp carnival, which would form an endowment for the education of a girl to be nominated by that Corps. These munificent contributions bear significant testimony to the liberality of our seamen and marines, and to their high opinion of the Home. The number of girls now in the Home is 154, and there are 39 boys being maintained and educated at Swanley at an annual cost of £900.

### THE REMOVAL OF SCARLET FEVER PATIENTS.

An unfortunate occurrence in Perthshire has been the subject of a report to the Scottish Local Government Board, and has attracted a good deal of attention and comment in the neighbourhood. Two children resident at Hill of Drip were certified to be suffering from scarlet fever and to be fit for removal to the Perth County Fever Hospital. They were accordingly put into the county ambulance waggon, a horsed vehicle, and died on the way. It turns out that they had malignant scarlet fever, which was the cause of death; but although their death is not attributed to the fact of removal, it is the opinion of the Medical Officer of Health for Perthshire that the children were not, in fact, in a fit state to be removed. As a result of the discussion which the case has aroused, it has been suggested that a motor ambulance should replace the horse-drawn one, with a view to the speedier removal of future patients. Whether this is practical politics at the moment is a matter for the local authorities concerned; but with another suggestion that has been made outsiders can legitimately express agreement. This is the proposal that by reciprocity among local hospitals serious cases likely to be prejudiced by removal over long distances should be admitted to the nearest hospital, not necessarily that to which the patient would in the ordinary course of events be admissible.

### A HOUSING PROPOSAL AT NEWCASTLE.

Dr. Harold Kerr, medical officer of health for Newcastle, who has often been concerned about the congestion and overcrowding of the city, and the scarcity of houses which helps to maintain them, has put forward the suggestion that temporary buildings on permanent foundations should be put up. There can be little question that such buildings, erected under proper supervision, would help to supply the present shortage. It is also evident that less initial expense would be incurred. We are not, however, convinced that cheap houses are the whole solution of the housing question. It is not sufficient that habitable dwellings should be built at small cost. The need is rather for a rigorous enforcement of the by-laws under which slum houses are condemned, and the building, if necessary, out of municipal funds, of suitable houses to replace them. The important thing is to raise the minimum standard of sanitation and fitness, so that at all events no house shall be tolerated which deserves the name of "slum." In aiding in this work the medical officers of health are faced with many difficulties, and sometimes corrupt interests; but the point is that the raising of the minimum allowed at all is at least as important as the provision of new dwellings of cheaper construction.

### GOVERNMENT DEDUCTIONS DURING HOSPITAL TREATMENT.

The Army Council has decided that, in view of the regulations which cancel the payment of separation allowance to any soldier's dependant in receipt of treatment at a rate-supported institution, local authorities must continue to notify the admission of soldiers' dependants to their institutions. At the meeting, at Stirling, of the county central district committee at which this decision was reported by the clerk who had been in correspondence with the Local Government Board on the subject, letters were read making strong complaint against two alleged cases, in which a soldier's wife was mulcted of all her separation allowance when in hospital, and when her child was in hospital had the allowance reduced by 4s. a week, although the allowance in respect of the child was only 2s. It was remarked, too, that it was the Government, and not the district which maintains the hospital, that received the money. It was decided to communicate with the Member for the constituency, and it is to be hoped that the matter will not be allowed to rest, for deductions during hospital treatment are difficult to justify, and if they are to be made the Government has far less claim than the soldier's wife or the hospital to them.

### EXPLOITATION OF V.A.D.S BY NURSING HOMES.

In a recent issue of The Hospital (October 14, 1916, page 43) attention was drawn to allegations by a doctor of wrongful exploitation of patriotic V.A.D. ladies by the managers of unspecified nursing homes. That these allegations are, at least to some extent, true, is the assurance of an anonymous correspondent, who tells us that the unpaid V.A.D. ladies at one nursing home even do much work for the private patients paying high fees. As a general rule, anonymous correspondents are rightly distrusted by practical people; but in this particular case it is obvious that the only inside information that is likely to be made available will be anonymous, and the usual rule against this sort of evidence must be relaxed. Our correspondent drops a vague hint as to the identity of the home referred to in her letter; and it is to be hoped that the state of things she discloses is confined to the establishment in question. On the nature and extent of this information it is impossible for an unofficial person, such as the Editor of The Hospital, to move in the matter. Our correspondent would do well to lay her knowledge before the War Office authorities, directly or indirectly; for if her charges are true, a grave public scandal is being perpetrated.

### THE SEGREGATION OF WOUNDED CANADIANS.

Our own views upon the proposals to segregate wounded Canadians in hospitals reserved for them alone were sufficiently expressed in a recent issue (The Hospital, October 14, 1916; page 20) and there is no need to repeat them. In fairness to those who have put forward the proposals in question, it should be added that there is something to be advanced in favour of them; though we are still unconvinced of the expediency and desirability of segregation in principle. Thus "A Canadian" states in the *Evening Standard* that the wounded soldiers themselves prefer to be in Canadian hospitals; not because they do not appreciate the skill and kindness with which they are treated in English hospitals, but simply from sentimental reasons. We feel bound to disagree with some of the statements of "A Canadian"; but this particular one may very possibly be accurate. In any case, it is clear that opinion in Canada is more favourable to the segregation of Canadian wounded than it is in England; for a recent telegram from Ottawa states that this is one of the reforms indicated in a sweeping report drawn up for the reorganisation of the Canadian Army Medical Service by Colonel Bruce, the Special Inspector-General of Medical Services.

### A HOSPITAL NURSE'S CLAIM FOR A LEGACY.

A curious case in which a trained nurse unsuccessfully claimed a legacy under the will of a patient was decided on October 20 by Mr. Justice Eve. The facts were that a lady was attended for twelve days before her death by a trained nurse. After her decease it was found that she left in her will certain legacies to her servants, by name.

Then followed a clause allocating "to each of my servants a further sum equal to their respective wages for one year." On the strength of this the nurse claimed £115 odd, and apparently would have succeeded in making good the claim had it not been for the presence of the word "further" in the sentence quoted above. The Judge found that the nurse was at the time of the testator's death one of her "servants"; but he thought that the true construction was that only the already named servants were intended to be entitled to the "further" legacies of a year's wages each, and that the nurse was not legally entitled to share their good fortune because she was not named specifically. Morally, the nurse's claim was certainly of the flimsiest; but this would not, of course, have debarred her from obtaining the legacy had the legal construction of the will been favourable to her claim.

### AN UNUSUAL TEST.

Ought candidates for the post of health visitor to be called upon to answer questions as to their personal practice in regard to alcoholic beverages? The point was raised at a meeting of the Neath Rural District Council last week, when a number of candidates appeared for an interview. The chairman said he had been handed a question which a member wished put to the applicants. It was, "Are you a total abstainer?" Another member, Mr. Henry Lewis, at once declared that the question was not a fair one to put, and said that if he were asked it he should decline to answer. A chorus of approval greeted this protest, with which Mr. E. J. Hopkins, "although a temperance man himself," expressed his entire agreement. The upshot of the incident was that the candidates escaped an inquiry on the line proposed.

### A PROBLEM OF NATIONAL HEALTH IN GREATER LONDON.

It is gratifying to learn that the London County Council is not going to delay in carrying into effect the recommendations of the Royal Commission on Venereal Diseases, and that facilities for treatment are to be provided at the earliest possible moment. The problem of arranging these facilities throughout Greater London is a most difficult and complicated one, but it augurs well for the success of the scheme in hand that the Lord Mayor convened a special conference of municipal and hospital authorities and representatives of the principal religious, educational, and social organisations to meet at the Mansion House on October 24. Addresses were given by the Home Secretary, the President of the Local Government Board, the Chairman of the London County Council, and the President of the National Council for Combating Venereal Diseases. The arrangements for the meeting were in the hands of the National Council for Combating Venereal Diseases, Kingsway House, Kingsway, W.C.

### NO ECONOMY IN HEALTH SERVICES.

The Local Government Board is circularising the local authorities, urging full provision for maternity and child welfare work, notwithstanding the need for economy in other directions, and intimating that the Board regards the work of health visiting as the most important element in any scheme of maternity and child welfare. The L.C.C., too, has been considering questions raised by the serious depletion which, consequent upon the war, has taken place in the sanitary staffs of some of the London Borough Councils, and suggesting that, having regard to the importance of maintaining an efficient Public Health Service, if male inspectors cannot be obtained as substitutes for those on war service, women should be employed. In a communication to the Lambeth Borough Council, confirming the appointment of a new health visitor, the Local Government Board calls upon the Council to consider at an early date the question of appointing two more.

### POLLING BOOTHS IN VOLUNTARY HOSPITALS.

The administrative side of voluntary hospital work has had many new experiences during the war, and one of the latest and most novel is that in connection with the working of the Military Service Referendum Act, 1916, of the Commonwealth of Australia. If amongst the wounded and sick soldiers, or included in the nursing staff, at our hospitals there are at present persons enrolled or entitled to be enrolled as electors of the Commonwealth, these persons have been given facilities for voting in the Referendum between October 16 and 28. The officer commanding the hospital automatically becomes an assistant returning officer, and it is his duty to appoint an "authorised witness" and a polling clerk, and to make efficient arrangements, under prescribed regulations, for the secrecy of the ballot. We are told that in one voluntary hospital a soldier requested and received instruction as to the meaning of the Referendum. He translated the explanation into his own language thus, "I am to say if those that haven't come out to do their bit shall be obliged to get a move on." There is very little doubt as to how this particular voter intends to mark his paper.

### THIS WEEK'S DRUG MARKET.

Few noteworthy price changes have occurred since last report appeared. As was anticipated there has been a substantial reduction in quotations for citrates following on the steady decline in the value of citric acid. Synthetic drugs are for the most part unchanged in price; salicylic acid and sodium salicylate are, however, a trifle lower; aspirin is in steady demand at unchanged rates; phenacetin is still very scarce and dear. Caffeine still fetches prices far above makers' nominal quotations. Castor oil has an upward tendency in value. Almond oil is slightly cheaper. Scammony root is getting very scarce. Liquid paraffin of pharmacopœial quality is more freely available than was the case a short time back. At the recent public sale of drugs in Mincing Lane, senna leaves, rhubarb, and honey realised lower prices; ipecacuanha, kola nuts, and Cape aloes tended lower, but the values of sarsaparilla and gamboge were well maintained.

